# Similarities and variances in perception of professionalism among Saudi and Egyptian Medical Students

**DOI:** 10.12669/pjms.326.11319

**Published:** 2016

**Authors:** Kamran Sattar, Sue Roff, Sultan Ayoub Meo

**Affiliations:** 1 Kamran Sattar, Department of Medical Education, College of Medicine, King Saud University, Riyadh, Saudi Arabia; 2 Sue Roff, Center for Medical Education, University of Dundee, Scotland; 3 Sultan Ayoub Meo, Department of Physiology, College of Medicine, King Saud University, Riyadh, Saudi Arabia

**Keywords:** Professionalism, Perception, Recommended Sanctions, Similarities, Variances

## Abstract

**Background & Objective:**

Professionalism has a number of culturally specific elements, therefore, it is imperative to identify areas of congruence and variations in the behaviors in which professionalism is understood in different countries. This study aimed to explore and compare the recommendation of sanctions by medical students of College of Medicine, King Saud University (KSU), Riyadh, Saudi Arabia and students from three medical colleges in Egypt.

**Methods:**

The responses were recorded using an anonymous, self-administered survey “ Dundee Polyprofessionalism Inventory I: Academic Integrity”. In the study 750 medical students of College of Medicine, KSU, Riyadh were invited and a questionnaire was electronically sent. They rated the importance of professionalism lapses by choosing from a hierarchical menu of sanctions for first time lapses with no justifying circumstances. These responses were compared with published data from 219 students from three medical schools in Egypt.

**Results:**

We found variance for 23 (76.66%) behaviors such as “physically assaulting a university employee or student” and “plagiarizing work from a fellow student or publications/internet”. We also found similarities for 7 (23.33%) behaviors including “lack of punctuality for classes” and drinking alcohol over lunch and interviewing a patient in the afternoon”, when comparing the median recommended sanctions from medical students in Saudi Arabia and Egypt.

**Conclusion:**

There are more variances than congruence regarding perceptions of professionalism between the two cohorts. The students at KSU were also found to recommend the sanction of “ignore” for a behavior, a response, which otherwise was absent from Egyptian cohort.

## INTRODUCTION

Professionalism has been recognized as a core characteristic of the medical profession and highly acknowledged as a fundamental component of clinical competency. Medical professionalism is a culture-sensitive construct, perceived and expressed concerning the inherent customs, beliefs and cultures.^1^,^2^ The quality of healthcare services offered by a physician has been declining in some parts of the globe.^3^ Medical professionalism is a compound societal model and geographical settings and culture play an essential role in any argument of professional behavior. Regional likenesses and differences are found in defining the professionalism due to the existing cultural differences.^1^,^4^ Additionally, it is acknowledged in literature that there is no predominant theoretical settings of medical professionalism currently universally recognized.^5^ Professionalism is considered ‘culture-sensitive’ and efforts are made to conceptualize professionalism in accordance to the Arabian context and recognizing it as an essential constituent of medical education^6^.

Worldwide the Dundee Poly Professionalism Inventory-I has been used to assess the professionalism related to academic integrity. The Dundee online inventory has been validated in the United Kingdom (UK) where data from two UK medical schools and a national reference group of medical educators demonstrate broad areas of agreement between students and faculty members on appropriate sanctions and responses to lapses in medical professionalism at the undergraduate students level. The Dundee Poly-professionalism inventory has questions that explore perceptions of the faculty and the students on the most frequent areas of concern related to student fitness to practice.^7^

Keeping in view the significance of professionalism, the present study aimed to explore the responses of medical students at the College of Medicine, King Saud University, Riyadh, KSA, in recommending sanctions for unprofessional behavior using “the Dundee Polyprofessionalism Inventory I: Academic Integrity”. We compared these responses among the students of College of Medicine, King Saud University, and the published data of Egyptian medical students’ responses collected using the same inventory. The present study also highlights the implication of cultural and social factors on the judgment of students on approving sanctions on professional lapses.

## METHODS

The present cross-sectional study was conducted in the Department of Medical Education, College of Medicine, King Saud University, Riyadh, Saudi Arabia during the academic year 2015-2016. Medical students’ perceptions of professionalism lapses were explored by asking the participants at King Saud University (KSU) to select from a “hierarchical menu of sanctions for first time lapses with no justifying circumstances by the medical students at undergraduate level and were compared with published data from Egypt using the Dundee Polyprofessionalism Inventory I: academic integrity.^7^

### Study Instrument

An anonymous, self-administered, bilingual (Arabic and English) “inventory (Dundee Polyprofessionalism Inventory I: academic integrity)” was administered through Bristol Online Survey system. The participants were asked to recommend the sanctions (Table-I), based on Teplitsky report.^8^

**Table-I:** *Hierarchy of recommended sanctions.

1.	“Ignore (None)”
2.	“Reprimand (verbal warning)”.
3.	“Reprimand (written warning)”
4.	“Reprimand, plus mandatory counseling”
5.	“Reprimand, counseling, extra work assignment”
6.	“Failure of specific class/remedial work to gain credit”
7.	“Failure of specific year (repetition allowed)”
8.	“Expulsion from college (readmission after one year possible)”
9.	“Expulsion from college (no chance for readmission)”
10.	“Report to a regulatory body”

### Participants

### College of Medicine, King Saud University

In the present study, initially 753 respondents were invited, three students declined to participate in the study: Out of 750, 162 (22%) were first-year medical students; 195 (26%) second-year; 160 (21%) third-year; 114 (15%) fourth-year; and 122 (16%) fifth- year students. Of the total agreed participants (n= 750), there were 441 (58.57%) males and 311 (41.30%) were females and 1(0.1) preferred not to say. There were 166 (22%) students from 17-19 years of age, 518 (68.8%) from 20-24, 69 (9.2%) from 25 or over.

### Egyptian Medical Schools

Of the total agreed participants (n=219), there were 125 (57.1%) males and 94 (42.9%) were female students. Of the total, there were 57 (26%) from 17-19 years of age, 124 (56.6%) from 20-24, 37(16.9%) from 25 or over and 1(0.5%) preferred not to say. The year of study was not reported for the Egyptian students.

### Ethics Approval and Participants Consent

The Institutional Review Board, College of Medicine, King Saud University approved the study. We also obtained consent from all the participants for the publication of the study.

### Data collection and analysis

The data were stored in a secured computer, the coded data were computed using the Microsoft Excel software and analyzed using SPSS version 21.0 statistical software; the comparisons as the median of students’ from KSA and Egypt responses were included.

## RESULTS

The participants’ response comparison as median recommended sanctions for first time lapses in 30 different kinds of professionalism with no justification in situations by undergraduate students in medical school are shown in Table II and III. It was noted that the students at KSU were found to select ignore as a recommended sanction for “Exchanging information about an exam before it has been taken (e.g. OSCE), whereas, Egyptian students recommended a sanction, Reprimand (verbal warning)”.

**Table-II:** Format of students responses to inventory statements

a) Is this wrong		
Yes	No	Unsure
b) “Do you think your fellow students do this”		
Yes	No	Unsure
c) “Have you ever done this in your present course”		
Yes	No	Unsure
d) “Would you ever do this in your present course”		
Yes	No	Unsure
e) “What level of sanction (1-10) should apply for a first time offence with no mitigating circumstances?		

* Hierarchy of recommended sanctions”.

**Table-III:** Demographic Characteristics of Medical Participants.

KSU n=750					3 Egyptian Medical Schools n=219			

Gender	Male	Female	Prefer not to say		Male	Female	Prefer not to say	
	441(58.57%)	311(41.30%)	1(0.1%)	125(57.1%)	94(42.9%)	0	

Age	*17- 19 Years*	*20- 24 Years*	*25 or over*	*Prefer not to say*	*17-19*	*20-24*	*25 or over*	*Prefer not to say*

	166(22%)	518(68.8%)	69(9.2%)	0	57(26.0%)	124(56.6%)	37(16.9%)	1(0.5%)

*Year of* Study	*1ST*	*2nd*	*3rd*	*4th*	*5th*			

	162(22%)	195(26%)	160(21%)	114(15%)	122(16%)			

Similarity in recommended sanctions between KSU students and the Egyptian students for 7 behaviors are shown in Table-IV : Lack of punctuality for classes. Sanction =Reprimand (verbal warning); Not doing the part assigned in group work. Sanction=Reprimand (written warning); Examining patients without knowledge or consent of supervising clinician. Sanction=Reprimand (written warning); Inventing extraneous circumstances to delay sitting an exam. Sanction=Reprimand (written warning); Purchasing work from a fellow student or internet etc. supplier. Sanction=Reprimand, plus mandatory counseling; Threatening or verbally abusing a university employee or fellow student. Sanction=Reprimand, counseling, extra work assignment; and Drinking alcohol over lunch and interviewing a patient in the afternoon. Sanction=Failure of specific class/remedial work to gain credit.

**Table-IV:** Similarity in response as median recommended sanctions among KSU and Egyptian medical students similarity in recommended sanctions as median for unprofessional behavior.

Survey Statements	KSA n=750	Three Egyptian Medical schools n=219
“Lack of punctuality for classes”	2	2
“Not doing the part assigned in group work”	3	3
“Examining patients without knowledge or consent of supervising clinician”	3	3
“Inventing extraneous circumstances to delay sitting an exam”	3	3
“Purchasing work from a fellow student or internet etc. supplier”	4	4
“Threatening or verbally abusing a university employee or fellow student”	5	5
“Drinking alcohol over lunch and interviewing a patient in the afternoon”	6	6

Three behavors for which higher sanctions were recommended by Egyptian students as compared to KSU students is shown in Table-V. “Reprimand, counseling extra work assignment”, was the sanction by KSU students for behavior [i] “intentionally falsifying test results or treatment records in order to disguise mistakes” whereas, the Egyptian students recommended higher sanction, i.e. “failure of specific class/remedial work to gain credit”. Similarly, one level higher sanction was observed for the behavior [ii] “sexually harassing a university employee or fellow student” where Saudi students recommended, “failure of specific year (repetition allowed)” but Egyptian students’ recommendation was “expulsion from the college (readmission after one year possible)”. Another behavior was [iii] “cutting and pasting or paraphrasing material without acknowledging the source” for which, again Saudi students were one level lenient and selected the sanction “reprimand (written warning)” while the Egyptian students’ selected for sanction “reprimand, plus mandatory counseling”.

**Table-V:** Variance in response as median recommended sanctions among KSU and Egyptian medical students.

“Getting or giving help for course work, against a teacher’s rules (e.g. lending work to another student to look at)”	3	2
“Removing an assigned reference from a shelf in the library in order to prevent other students from gaining access to the information in it”	4	3
“Signing attendance sheets for absent friends, or asking classmates to sign attendance sheets for you in labs or lectures”	2	3
“Exchanging information about an exam before it has been taken (e.g. OSCE)”	1	2
“Forging a healthcare worker’s signature on a piece of work, patient chart, grade sheet or attendance form”	5	4
“Claiming collaborative work as one’s individual effort”	4	2
“Altering or manipulating data (e.g. adjusting data to obtain a significant result)”	4	6
“Failure to follow proper infection control procedures”	4	6
“Attempting to use personal relationships, bribes or threats to gain academic advantages by e.g. getting advance copies of exam papers or passing exam by such pressures on staff”	5	4
“Engaging in substance misuse (e.g. drugs)”	5	8
“Completing work for another student”	3	2
“Intentionally falsifying test results or treatment records in order to disguise mistakes”	5	6
“Physically assaulting a university employee or student”	5	8
“Providing illegal drugs to fellow students”	7	9
“Sabotaging another student’s work”	5	7
“Sexually harassing a university employee or fellow student”	7	8
“Resubmitting work previously submitted for a separate assignment or earlier degree”	3	6
“Plagiarizing work from a fellow student or publications/internet”	4	7
“Cheating in an exam by e.g. copying from neighbor, taking in crib material or using mobile phone or getting someone else to sit for you”	5	7
“Cutting and pasting or paraphrasing material without acknowledging the source”	3	3.50
“Damaging public property e.g. scribbling on desks or chairs”	4	8
“Falsifying references or grades on a curriculum vitae or altering grades in the official record”	5	7
“Involvement in pedophilic activities - possession/viewing of child pornography images or molesting children”	7	9

### Egyptian students were at-least two levels stricter in their sanctions than the Saudi students

Egyptian students recommended two levels stricter sanctions (Table-V) than that of Saudi students for: [i] “Altering or manipulating data (e.g. adjusting data to obtain a significant result). [ii] Cheating in an exam by copying from neighbor, taking in crib material or using mobile phone or getting someone else to sit for you. [iii] Falsifying references or grades on curriculum vitae or altering grades in the official record. [iv] Involvement in pedophilic activities possession/viewing of child pornography images or molesting children and [v] altering or manipulating data e.g. adjusting data to obtain a significant result”.

Moreover, the difference of sanctions among the both cohorts, increases further as it was evident from Table-V that the sanction approved by Egyptian students was three levels stricter than that of Saudi students, for the 4 behaviors: [i] “Resubmitting work previously submitted for a separate assignment or earlier degree. [ii] Plagiarizing work from a fellow student or publications/internet. [iii] Physically assaulting a university employee or student and [iv] engaging in substance misuse e.g. drugs”.

The students from Egypt recommended four level higher sanction for the behavior, “Failure to follow proper infection control procedures “as they recommended “Failure of specific class/remedial work to gain credit” while KSU students recommended “Reprimand (verbal warning).”

### Saudi students were at-least one level strict in their sanctions than the Egyptian students

For the following two behaviors the recommendation from Saudi students was stricter than that of Egyptian students: [i] “Completing work for another student” and [ii] attempting to use personal relationships, bribes or threats to gain academic advantages by getting advance copies of exam papers or passing the exam by such pressures on the staff”.

It was also noted that the students from Egypt recommended more lenient sanctions for, “Claiming collaborative work as one’s individual effort”. Their recommendation was Reprimand (verbal warning) which was two levels lower than that from KSU where the selected recommendation was Reprimand, plus mandatory counseling.

## DISCUSSION

The present study was carried out to elucidate the recommendations on sanctions for unprofessional behavior meted out by students of the College of Medicine, KSU, Riyadh, Saudi Arabia. The responses were compared with the published data^9^ from Egyptian Medical School carried out using the same inventory as used in the present study.

It was found that variances were more than the congruence in the responses of the respondents’ cohorts. Such differences were also reported^7^ for recommended sanctions by the students from a Scottish medical school. The period of study at medical school is the foundation stone for ethical and moral values for the future physicians. According to another study^10^ the medical students who showed unprofessional behavior in medical school were more expected to have consequent state board corrective actions. In the literature, researchers^11^-^13^ suggest that the professionalism should be taught and assessed in a way that it should address cognitive and behavioral outcomes.

Similarly, a study was conducted using the same inventory and reported that 54% of the students recommended sanctions in Scottish medical school about lapses in academic integrity.^7^ Serious problems allied to academic integrity in Pakistan were recognized that require timely solutions.^14^ The revelation of present study is in agreement with the above findings. Our study demonstrats that cheating is not confined to examination such as “copying from neighbor, taking in crib material or using mobile phone or getting someone else to sit for you, but also included lapses in academic integrity such as Signing attendance sheets for absent friends, or asking classmates to sign attendance sheets for you in labs or lectures and resubmitting assignments work previously submitted for a separate assignment”.

The collection and comparison of the participants’ perception of medical professionalism enabled to determine the prevalence of professionalism lapses linked to academic integrity by students in the College of Medicine, KSU. Which in turn helps to recognize where the mediation and further strengthening of professionalism teaching are mandatory. The results explain the implication of cultural and social aspect of the country on recommending sanctions for professional lapses. Additionally, it indicates an urgent need to improve the certain areas of professional activity irrespective of their cultural affiliation. Hence, it is imperative to endorse and spread the values of academic integrity and professional behaviors by enhanced teaching methods and implement certain regulations on Polyprofessionalism to help the students.

This information, in turn, can be used to mark and additionally refine the medical education aligned with expected values of professionalism and ultimately it will enhance the standards of health care services. It also enables College of Medicine, KSU curriculum planners to identify where interventions regarding the teaching of professionalism are required.

### Study Limitations

The present study has been limited to testing the feasibility of an online inventory to ‘map’ student understanding of the relative importance of various lapses in academic integrity through the ‘proxy’ of soliciting recommended sanctions. There may be a response bias among those responders versus non-responders.

## CONCLUSION

In this study, few issues have been identified related to the academic integrity as students preferred to opt for ignore sanctions which amounts to lapse in academic integrity and professional behavior. The outcome of the study indicates that the medical students had a reduced thoughtfulness of the significance of some lapses of professionalism relating to academic integrity. This necessitates urgent intervention by the teaching fraternity to help them understand the impact of such a perception of their professional integrity. Strong adherence to national culture makes medical students culturally specific, but it is the need of the hour to have educational and training modalities to be analysed.
